# *UL56* Is Essential for Herpes Simplex Virus-1 Virulence In Vivo but Is Dispensable for Induction of Host-Protective Immunity

**DOI:** 10.3390/vaccines12080837

**Published:** 2024-07-25

**Authors:** Nopprarat Tongmuang, Meera Krishnan, Viv Connor, Colin Crump, Liselotte E. Jensen

**Affiliations:** 1 Department of Microbiology, Immunology and Inflammation, Temple University Lewis Katz School of Medicine, Philadelphia, PA 19104, USA; 2 Center for Inflammation and Lung Research, Temple University Lewis Katz School of Medicine, Philadelphia, PA 19104, USA; 3 Department of Pathology, University of Cambridge, Cambridge CB2 1QP, UK; 4 Cancer Signaling and Microenvironment, Fox Chase Cancer Center, Temple Health, Philadelphia, PA 19111, USA

**Keywords:** herpes simplex virus, HSV, IL-1, IL-1R1, IL-1RAP, IL-1RL2, IL-36, immune memory, UL56

## Abstract

Herpes simplex virus-1 (HSV-1) is common and can cause significant disease in humans. Unfortunately, efforts to develop effective vaccines against HSV-1 have so far failed. A detailed understanding of how the virus infects its host and how the host mounts potent immune responses against the virus may inform new vaccine approaches. Here, using a zosteriform mouse model, we examined how the HSV-1 gene *UL56* affects the ability of the virus to cause morbidity and generate protective immunity. A *UL56* deletion mutant, ΔUL56, was derived from the wild-type HSV-1 strain SC16, alongside a revertant strain in which *UL56* was reintroduced in ΔUL56. In vitro, the three virus strains replicated in a similar manner; however, in vivo, only the wild type and the revertant strains caused shingles-like skin lesions and death. Mice previously infected with ΔUL56 became resistant to a lethal challenge with the wild-type SC16. The protective immunity induced by ΔUL56 was independent of IL-1, IL-33, and IL-36 signaling through IL-1RAP. Both skin and intramuscular ΔUL56 inoculation generated protective immunity against a lethal SC16 challenge. After 6 months, female mice remained resistant to infection, while male mice exhibited signs of declining protection. Our data demonstrate that *UL56* is important for the ability of HSV-1 to spread within the infected host and that a ∆UL56 strain elicits an effective immune response against HSV-1 despite this loss of virulence. These findings may guide further HSV-1 vaccine development.

## 1. Introduction

Herpes simplex viruses (HSV-1 and HSV-2) are common human pathogens that cause a spectrum of diseases from minor afflictions such as oral and genital sores to grave conditions like encephalitis. During a primary infection with HSV-1, the virus establishes life-long infections of trigeminal and dorsal root ganglia. The virus typically enters a latent state during which no disease is apparent. Upon mild or significant immune suppression of the host, the virus begins to replicate and then spreads to other sites where it causes disease within the host. In skin and mucosal sites, the virus specifically replicates in keratinocytes. As the host’s immune response is engaged, the active infection is cleared, and the virus reenters latency in the ganglia. The 152 kbp HSV-1 genome encompasses approximately 80 genes, each known or presumed to have a pivotal function in the virus’s life cycle or pathogenicity. Many of the encoded proteins are part of immune evasive strategies deployed by the virus. These factors are often considered promising targets for the development of preventive and therapeutic vaccines. However, despite extensive research, effective vaccines against HSV remain elusive.

While many of the HSV-1 genes have been comprehensively studied, others have received less attention. One gene that remains poorly understood is *UL56*, which encodes the protein pUL56. Recent in vitro work has implicated pUL56 in immune system evasion by suppressing the engagement of innate pathogen sensors and cytokine signaling [1,2,3], possibly through the modulation of ubiquitin ligases [4]. In a sub-strain-dependent manner, UL56 may also affect the ability of HSV-1 to spread from cell to cell through the formation of syncytia [5]. Earlier in vivo work in mice revealed that *UL56* is not required for viral replication or establishment of latent ganglion infections [6,7]. However, deletion of *UL56* did impact pathogenicity in intra-nasal and -peritoneal mouse models [2,8]. Independently, bioinformatics analyses predicted the membrane-bound pUL56 to be a possible vaccine target [9].

An important function of vaccine adjuvants is to promote inflammasome activation that leads to the cellular release of IL-1 [10,11]. The IL-1 family represents a group of cytokines involved in the activation of both innate and adaptive immune responses, including those directed against viruses [12,13]. The most extensively studied member of the family is IL-1, which is well documented to activate dendritic and T cells in support of developing both cellular and humoral immunity [10,11]. During initial HSV-1 infection of keratinocytes, IL-1 is rapidly released by cells [14,15]. This IL-1 acts as a classical alarmin by activating dendritic cells and downstream adaptive immune mechanisms. However, during HSV-1 infections, IL-1 family cytokines can also promote the production of type I interferons (IFN-I) [15]. IFN-I regulate a plethora of genes, including many associated with innate antiviral mechanisms within the infected and neighboring cells [16,17]. IFN-I also provide further support for the adaptive immune system [18]. Thus, the IL-1 family has multifaceted functions aimed at promoting rapid and effective immune responses against HSV-1 and many other viruses.

In this study, we set out to evaluate if pUL56 contributes to HSV-1 evading IL-1-family-dependent immune responses in the skin. Using a mutant HSV-1 virus lacking *UL56* and a flank skin HSV-1 infection mouse model, we examined disease progression in knockout mice unable to respond to the IL-1, IL-33, and IL-36 cytokines due to lack of either the IL-36 receptor, IL-1RL2, or the common, for all three cytokines, receptor co-factor, IL-1RAP. The performed experiments reveal a critical role of *UL56* in HSV-1 disease progression. The data also demonstrate that potent protective immune responses against HSV-1 can be engaged in IL-1RL2- and IL-1RAP-independent manners.

## 2. Materials and Methods

### 2.1. Cloning of ΔUL56 Mutant and Revertant HSV-1 Strains

To generate SC16 lacking UL56, a knockout homologous recombination targeting plasmid was first generated containing a ~3.75 Kbp region of SC16 genomic DNA (region 114,423..118,177) with the UL56 CDS centrally located (region 115,941..116,645; numbers based on MN159383.1), where the entire UL56 coding region was replaced by the coding region for eGFP, followed by a single 3’ EcoRI site. Vero cells were co-transfected with this knockout plasmid and SC16-infected cell DNA and incubated until a visible cytopathic effect was observed. Limiting dilution was used to isolate a single GFP-positive plaque, and this was propagated in Vero cells to generate the SC16-∆UL56 stock. To generate a revertant virus, a rescue homologous recombination targeting plasmid was generated by replacing the eGFP coding region in the knockout plasmid with the authentic UL56 coding region. Vero cells were co-transfected with this rescue plasmid and SC16-∆UL56-infected cell DNA and incubated until a visible cytopathic effect was observed. Limiting dilution was used to isolate a single GFP-negative plaque, and this was propagated in Vero cells to generate the SC16-UL56R stock. All virus stocks were propagated and titrated using Vero cells and loss or recovery of pUL56 expression confirmed by SDS-PAGE following Western blot analysis.

### 2.2. Cell Cultures and In Vitro Infections

Human HaCaT epidermal keratinocytes (obtained from Dr. Meenhard Herlyn, Wistar Institute, Philadelphia, PA, USA) and Vero cells (ATCC, Manassas, VA, USA) were maintained in DMEM supplemented with 10% fetal bovine serum and Gentamicin. Vero cells were used for the production of the virus for in vitro and in vivo infections. Virus stocks were titrated on Vero cells to determine plaque forming units (PFUs).

For in vitro infections, HaCaT cells were seeded in black 96-well plates with clear bottoms. The cells were allowed to grow for 7 days after reaching confluence. During this period, the medium was replaced every 1–2 days. Cells were infected with HSV-1 for 1 h in serum-free DMEM. The inoculum-containing medium was replaced with serum-containing DMEM, and the infections were allowed to progress for 48 h.

### 2.3. Cell Staining, Imaging and Quantification

HaCaT cells were sequentially fixed and permeabilized in formaldehyde and 0.1% Triton X-100, respectively. Cells were blocked with Intercept (TBS) Blocking Buffer (Li-Cor, Lincoln, NE, USA). Cells were incubated in the same buffer supplemented with 0.2% Tween 20, HSV-1 Antibody (20.7.1) Alexa Fluor 790 (1/200, sc-57863 AF790, Santa Cruz, Santa Cruz, CA, USA) and CellTag 700 Stain (1/500, Li-Cor, Lincoln, NE, USA) for 2 h or overnight. Cells were washed 4 times in TBS, 0.2% Tween 20 and imaged using an Odyssey DLx digital fluorescence scanner (Li-Cor). Digitized scans were examined using EmpiriaStudio (Li-Cor) and HSV-1 near infrared (NIR) fluorescence signal (NIR 790) standardized against cell signal (NIR 700).

### 2.4. Mice and Genotyping

All work with mice was approved by the Temple University Institutional Animal Care and Use Committee under the Animal Welfare Assurance Number A3594-01 and in compliance with the United States Public Health Service Policy on Humane Care and Use of Laboratory Animals. C57BL/6J and C57BL/6N mice were obtained from the Jackson Laboratory (Bar Harbor, MA, USA). *Il1rl2^−/−^* mice (C57BL/6NCrl-Il1rl2^em1(IMPC)Mbp^/Mmucd, 050664-UCD, RRID:MMRRC_050664-UCD) were obtained from The Mutant Mouse Regional Resource Center (Davis, CA, USA). The strain background for this strain is C57BL/6N. *Il1rap^−/−^* mice [19] were obtained from the Jackson Laboratory (B6;129S1-Il1rap^tm1Roml^/J, #003284, RRID:IMSR_JAX:003284). To purify the genetic background, the strain was backcrossed to the C57BL/6J strain for 11 generations. Ear punches collected from pups were genotyped by denaturing them in 1 mM NaOH, 0.1 mM EDTA at 95 °C for 10 min. Samples were neutralized with Tris HCl pH 8.0. Genotyping was performed using the primers in Table 1 and standard PCR and gel electrophoresis. All experimental mice were bred in-house using homozygous breeders.

### 2.5. In Vivo Infections

Mice were infected with HSV-1 on the flank using a previously described method ([20] and refs. therein). Briefly, the mice were denuded through sequential use of an electrical trimmer and epilating cream. The following day, a drop containing 1.5 × 10^6^ PFU HSV-1 was placed on the flank, and the bevel of a needle was used to abrade the skin through the drop. The progression of skin infections was documented by photographing the mice next to a ruler. The latter was used for standardization to size lesions using the open software Image J (version 1.54j, https://imagej.net/ij/, accessed 24 July 2024). Mice that exhibited signs of severe lethargy, paralysis (one or both hind legs dragging), or greater than 20% weight loss were considered moribund and euthanized. For intramuscular infections, 1.5 × 10^6^ PFU ΔUL56 in 20 μL was injected into the right thigh muscle using insulin syringes. The mice were under isoflurane anesthesia for all procedures requiring restraint of the mice.

### 2.6. Statistical Analyses

All experiments were performed at least twice except for the long-term 6-month challenge experiment, which was only performed once. Data were graphed and analyzed for statistical significance using GraphPad Prism 10 (GraphPad, La Jolla, CA, USA). Survival data were evaluated using Log-rank and Gehan–Breslow–Wilcoxon tests. All other groups were compared using t tests. *p* values lower than 0.05 were considered significant.

## 3. Results

### 3.1. Generation of a UL56 Deletion Mutant, ΔUL56

**Figure 1:**
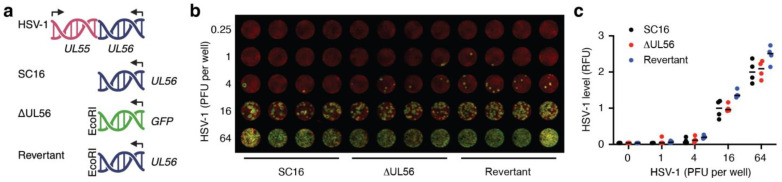
The *UL56* deletion mutant ΔUL56 retains the ability to replicate in keratinocytes. (**a**) A *UL56* deletion mutant, ΔUL56, was generated by replacing the *UL56* gene in the HSV-1 strain SC16 with the coding sequence for GFP. A revertant strain was also generated by reintroducing *UL56* in ΔUL56. (**b**,**c**) Keratinocytes were infected with SC16, ΔUL56, or revertant viruses as indicated. After 48 h, HSV-1 and cells were labeled with anti-HSV1-AF790 and CellTag 700. Stained cells were imaged (**b**), NIR signals were quantified, and the HSV-1 signal was standardized against CellTag (**c**).

Our groups have previously independently examined mechanistic aspects of how UL56 modulates cellular functions [1,3] and how the IL-1 cytokine family initiates immune responses ([14,20] and refs. therein) during HSV-1 infection of keratinocytes and the skin. We wanted to further examine the possible immune evasive properties of pUL56 in a zosteriform skin infection mouse model. This required a *UL56* deletion mutant derived from an HSV-1 strain with neurotropic properties in mice. Hence, we replaced the *UL56* gene in the HSV-1 strain, SC16, with the coding sequence for green fluorescent protein (GFP) using conventional homologous recombination between viral genomic DNA and a plasmid containing ~1.5 Kbp 5’ and 3’ flanking regions of the *UL56* locus from the SC16 genome placed either side of the GFP coding sequence (Figure 1a, ΔUL56). To further offset any potential impact of the introduced EcoR1 site (Figure 1a), we generated a revertant strain in which the *GFP* sequence in ΔUL56 was replaced, in a similar manner, with the SC16 *UL56* gene (Figure 1a, Revertant).

### 3.2. UL56 Replicates in Skin Keratinocytes In Vitro

To access the abilities of the two newly generated viruses to replicate in keratinocytes, the two mutants, ΔUL56 and revertant, and the wild-type SC16 strain were titrated on human HaCaT cells (Figure 1b,c). Both ΔUL56 and the revertant strain generated plaques (Figure 1b) and overall infections (Figure 1c) that were indistinguishable from those resulting from SC16 infections. Thus, the replication competence of the ΔUL56 and revertant mutants was retained in keratinocytes, i.e., *UL56* does not appear to be involved in the viral lifecycle at the single-cell level.

### 3.3. UL56 Has Diminished Capacity to Establish In Vivo Infections

**Figure 2:**
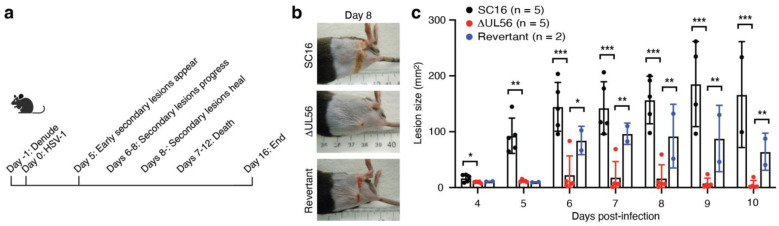
The mutant virus ΔUL56 results in fewer and smaller secondary skin lesions. (**a**) Timeline for a typical HSV-1 flank skin infection experiment. (**b**,**c**) C57BL/6J mice were infected with 1.5 × 10^6^ PFU SC16 (black bars in (**c**)), ΔUL56 (white bars in (**c**)), and the revertant (gray bars in (**c**)) HSV-1 strains on day 0. Skin lesions were photographed daily next to a ruler starting on day 4. Representative images of day 8 lesions are shown in (**b**). Using the imaged ruler for scale, lesion sizes were determined using the freehand selection tool in the Image J software. Each dot per time point represents a single mouse. *, *p* < 0.05; **, *p* < 0.01; ***, *p* < 0.001.

Active manifestations of HSV infection in vivo involve replication of the virus in skin keratinocytes; however, both innate and adaptive immune responses contribute to limiting the ability of the virus to propagate and spread. We therefore evaluated the capability of the ΔUL56 mutant to establish infections in a mouse zosteriform skin infection model. In this model, the virus is introduced into the flank skin through abrasion with a needle (Figure 2a). In this primary infection site, the virus replicates and enters sensory neurons. The virus then migrates retrograde to the dorsal root ganglion, where it further replicates. This is followed by viral anterograde migration through the dermatome into the skin, where the virus infects keratinocytes and causes secondary lesions that resemble varicella zoster recrudescence in shingles (Figure 2a). This typical pattern of disease progression was observed when C57BL/6 mice were infected with either the wild-type SC16 or the revertant HSV-1 strains (Figure 2b,c). However, in mice infected with the ΔUL56 mutant, secondary lesions were rarely observed and, when present, were generally noticeably smaller than those caused by the wild-type and revertant viruses (Figure 2b,c). In these virus comparative studies, mice infected with either SC16 or the revertant started to become moribund at days 9–10, and the experiments were therefore stopped. These data demonstrate that the ΔUL56 mutant is significantly attenuated in vivo and further suggests that pUL56 is essential for HSV-1 to establish infections in vivo.

### 3.4. UL56 Induces Protective Immunity against a Lethal Challenge with Wild-Type Virus

**Figure 3:**
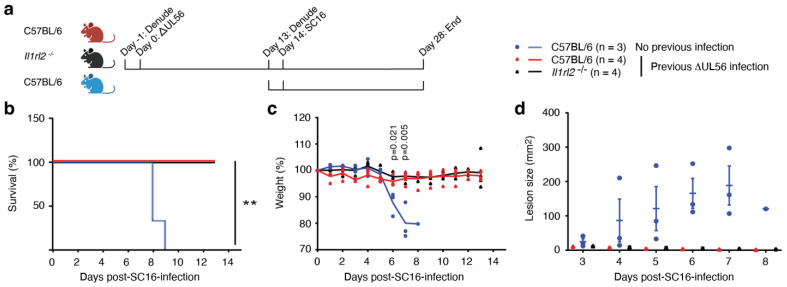
Previous exposure to ΔUL56 provides protection from a lethal challenge with wild-type SC16 HSV-1 in C57BL/6 and *Il1rl2^-/-^* mice. (**a**) Experimental timeline. Wild-type C57BL/6N and *Il1rl2^-/-^* male mice were infected with 1.5 × 10^6^ PFU ΔUL56 on the right flank. After 14 days, the mice and a C57BL/6N control group were infected with 1.5 × 10^6^ PFU SC16 on the left flank. Survival (**b**), weight (**c**), and lesions (**d**) were monitored for an additional 13 days. (**c**) The indicated *p* values compare wild-type mice with and without prior ΔUL56 exposure. **, *p* < 0.01.

Several lines of evidence from cell culture experiments indicate that an important function of pUL56 is to suppress one or more branches of the host immune system [1,2,3]. Thus, our findings, indicating that the ΔUL56 mutant has diminished infectivity in vivo (Figure 2) but not in vitro (Figure 1), could suggest that adaptive immune responses directed against HSV-1 are suppressed by pUL56; i.e., in the absence of *UL56,* stronger protective immune responses are engaged by HSV-1, and these prevent the appearance of secondary lesions. To test this, with the longer-term objective of examining the role of IL-1 related cytokines in memory responses, we infected mice with ΔUL56 on the flank (Figure 3a). Two weeks later, this initial infection was followed by a challenge with the wild-type SC16 HSV-1 strain (Figure 3a). The first and second infections were placed on opposite sides of the mice to mitigate possible effects of the inflammatory responses to the first infection. While, as expected, the mice that did not receive ΔUL56 succumbed to the SC16 infection within 9 days (Figure 3b, blue line), all the mice that had previously been exposed to ΔUL56 survived (Figure 3b, red line). Preexposure to ΔUL56 also prevented weight loss (Figure 3c) and the development of secondary lesions (Figure 3d). This demonstrates that potent protective immune responses are induced in mice, independent of *UL56*.

### 3.5. Protective Immunity Induced by ΔUL56 is Independent of IL-36 Signaling

Our previous studies involving the flank mouse model examined the role of IL-36 in initiating immunity against HSV-1 ([20] and refs. therein). Others showed that IL-36 can act as an adjuvant for immunotherapy [21] and vaccination against zika virus [22]. Furthermore, vaccination of female guinea pigs with a live attenuated HSV-2 strain VC2 promoted differential expression of IL-36 in the vaginal mucosa upon challenge with the pathogenic HSV-2 G strain [23]. Thus, for some time, we had been interested in delving deeper into the potential role of IL-36 in memory responses. However, the mortality in the model (Figure 2a) represented a significant challenge, as our IL-36 knockout mice died more frequently than wild-type control mice ([20] and refs. therein). The ability of the ΔUL56 mutant virus to induce protective immunity without causing mortality (Figure 3) represented an opportunity to revisit this area of research. Therefore, concurrent with the above-described challenge experiment, we also examined whether mice lacking IL-1RL2, the receptor for IL-36, as well as wild-type mice, would be protected from HSV-1 following ΔUL56 exposure (Figure 3). The *IL1rl2^−/−^* mice proved to be as resilient against the lethal SC16 challenge as the wild-type mice, as they all survived (Figure 3b), they did not lose significant weight (Figure 3c), and they did not develop any secondary lesions (Figure 3d). This suggests that IL-36 signaling is not essential for the development of protective immunity against a second infection with HSV-1.

### 3.6. IL-1RAP Is Not Required for Protective Immunity Engaged by ΔUL56

**Figure 4:**
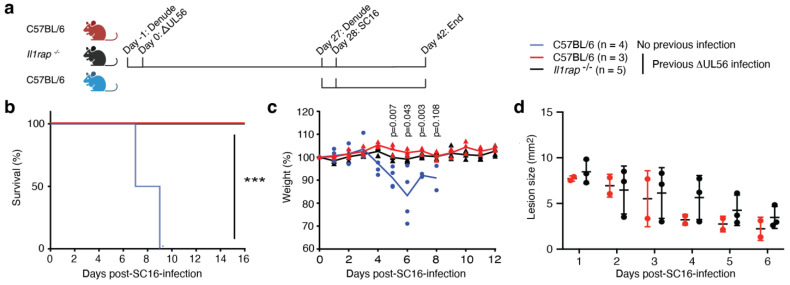
Wild-type and *Il1rap^−/−^* mice previously exposed to ΔUL56 are protected from a lethal SC16 challenge. (**a**) Experimental timeline. Wild-type C57BL/6J and *Il1rap^−/−^* male mice were infected with 1.5 × 10^6^ PFU ΔUL56 on the left flank. After 28 days, the mice and a C57BL/6 control group were infected with 1.5 × 10^6^ PFU SC16 on the right flank. Survival (**b**), weight (**c**), and lesions (**d**) were monitored for an additional 16 days. (**b**) ***, *p* < 0.001. (**c**) The indicated *p* values compare wild-type mice with and without prior ΔUL56 exposure. (**d**) Due to fur growth, not all secondary lesions could be quantified. Only mice previously infected with ΔUL56 are shown. No statistically significant differences between wild-type and *Il1rap^−/−^* were observed.

IL-36 is part of the IL-1 family of cytokines, which also includes the founding members IL-1α and IL-1β and IL-33. We and others have demonstrated the importance of IL-1 [14,15], IL-33 [24,25,26], and IL-36 ([20] and refs. therein) in the initiation of immunity against HSV. The IL-1, IL-33, and IL-36 cytokines all utilize IL-1RAP as a co-factor for receptor signaling [13]. We therefore wondered whether preventing signaling by all these cytokines would affect the protective immune response elicited by ΔUL56. Hence, we infected wild-type and *Il1rap^−/−^* mice with ΔUL56 (Figure 4a). One month later, these mice, and a control group that did not receive ΔUL56, were challenged with SC16. While both the wild-type and the *Il1rap^−/−^* mice that had previously received ΔUL56 survived (Figure 4b) and maintained their weight (Figure 4c), the control group lost weight and succumbed to the SC16 infection (Figure 4b,c). Furthermore, none of ΔUL56-exposed mice developed secondary lesions irrespective of their genotype (Figure 4d). No statistically significant differences between the ΔUL56-exposed wild-type and *Il1rap^−/−^* mice were detected (Figure 4b–d). This suggests that IL-1 family cytokine signaling through IL-1RAP is not required for the development of immune memory and protection against a second HSV infection.

### 3.7. Intramuscular Administration of ΔUL56 Provides Protective Immunity

**Figure 5:**
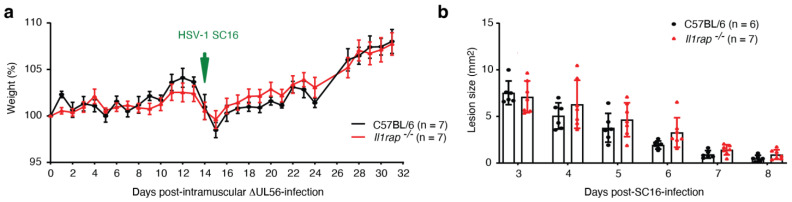
Following intramuscular administration of ΔUL56, wild-type and *Il1rap^−/−^* mice are protected from a lethal challenge with wild type SC16. Wild-type C57BL/6J and *Il1rap^−/−^* male mice were injected in the right thigh muscle with 1.5 × 10^6^ PFU ΔUL56. After 14 days, the mice were infected with 1.5 × 10^6^ PFU SC16 on the right flank. Survival, weight (**a**), and lesions (**b**) were monitored for an additional 18 days. Because of fur growth, lesions on one C57BL/6 mouse could not be evaluated. No statistically significant differences were observed between the two mouse strains.

IL-1 is classically viewed as a major player in orchestrating skin immunity [27], and we were somewhat surprised that knocking out IL-1RAP in mice did not affect the experimental outcome above (Figure 4). The route of antigen administration or pathogen exposure plays an important role in guiding the differentiation of memory T cells for homing in different organs; e.g., skin exposure results in skin homing tissue resident T cells, while gut challenges typically lead to T cells that reside in the intestines [27]. We therefore wondered if exposure of the mice to ΔUL56 through a route different than the skin would affect protection. Consequently, we injected wild-type and *Il1rap^−/−^*mice intramuscularly with ΔUL56 (Figure 5a). The mice showed no significant signs of disease from these injections (Figure 5a). Two weeks later, the mice were challenged with SC16 on the flank. Because of the severity of the SC16 infection in mice not previously exposed to ΔUL56 (Figure 3 and Figure 4), we stopped the inclusion of unvaccinated control groups to minimize animal distress. Vaccinated mice in neither group died nor developed secondary lesions (Figure 5). Note that the lesions quantified in Figure 5b are the primary lesions arising from the SC16 inoculation abrasions. We observed no statistically significant differences between the two strains. These findings confirm that the protection conferred by ΔUL56 exposure is independent of IL-1RAP and can be engaged through intramuscular inoculation.

### 3.8. Protective Immunity Elicited by ΔUL56 Lasts Longer in Female Mice

**Figure 6:**
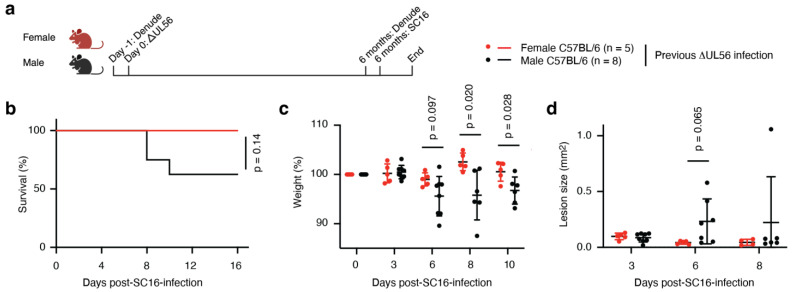
Female mice have more effective long-term protection against HSV-1 than male mice following ΔUL56 infection. (**a**) Experimental timeline. Female and male mice were inoculated with 1.5 × 10^6^ PFU ΔUL56 on the left flank. After 6 months, the mice were infected with 1.5 × 10^6^ PFU SC16 on the right flank. Survival (**b**), weight (**c**), and lesions (**d**) were monitored for an additional 16 days. (**c**,**d**) Note that because of death, two male mice were not evaluated starting at day 8. Furthermore, due to fur regrowth, lesion sizes could not be evaluated on one male and one female mouse starting at days 6 and 8, respectively.

Having established that ΔUL56 results in effective immunity, we further wanted to examine how long this protection lasts. Female and male mice were infected with ΔUL56 on the flank (Figure 6a). Six months later, they were challenged with a lethal SC16 dose (Figure 6a). All the female mice survived (Figure 6b) and exhibited no significant changes in weight (Figure 6c). They also showed no signs of developing secondary lesions (Figure 6d). By contrast, the male mice significantly lost weight (Figure 6c), and three proceeded to die (Figure 6b). Two of the males developed the classical zosteriform lesion band, while another two males developed very mild, but detectable, secondary lesions (Figure 6d). Thus, while the ΔUL56 mutant appears to elicit effective immunity lasting for months, the protection seems to decline more rapidly in male mice.

## 4. Discussion

Many of the over 80 genes encoded by the HSV-1 genome have well-defined functions, yet *UL56* remains poorly understood. Research in recent years has established intracellular immune evasion functions involving viral sensors and cytokine receptors and signaling pathways [1,2,3,4]. However, the significance of these mechanisms in vivo has not been determined. Here we show, using well-established cell culture and mouse models, that a mutant HSV-1 virus lacking *UL56,* ΔUL56, remains capable of replicating in cells (Figure 1) but becomes significantly attenuated in vivo (Figure 2). The weakened ΔUL56 virus induces potent protective immunity against lethal challenges with the wild-type HSV-1 strain when administered through skin abrasion (Figure 3) or intramuscular injections (Figure 5). The elicited immune response is long-lived (Figure 6) and independent of IL-1 cytokine family signaling (Figure 3, Figure 4 and Figure 5). Interestingly, female mice are protected by the ΔUL56 virus longer than male mice (Figure 6). Overall, our data demonstrate that *UL56* is important for HSV-1 virulence and dispensable for the induction of protective immunity in mice.

HSV infections in neonates can be life threatening. Fortunately, despite both HSV-1 and HSV-2 being wide-spread, such infections are extremely rare. The risk to the embryo or newborn is greatest when the mother is infected for the first time during the third trimester of a pregnancy [28]. This supports the view that it is possible to develop immunity in humans that can prevent infections, and thus, in theory, it should be possible to develop prophylactic vaccines [29]. Many different HSV-1 proteins have been evaluated over the years as vaccine targets, and several vaccine candidates are currently in different stages of clinical development. Glycoproteins present on the surface of the viral particle are considered good targets for neutralizing antibodies, while the deletion of intracellular viral immune evasion factors can be used to develop attenuated live vaccines with potential to elicit T cell responses [29,30]. The safety of the latter may be improved by eliminating, for example, UL37-directed neuroinvasion [31].

Bioinformatic approaches identified pUL56 as a possible vaccine target [9]. Here, our data demonstrate that potent protective immunity develops in response to an HSV-1 mutant that lacks *UL56* (Figure 3, Figure 4, Figure 5 and Figure 6). While these findings contribute to defining the minimal immunogenic HSV-1 viral particle, it does not imply that T cells directed against pUL56 may not have protective activities. The fact that *UL56* is involved in immune evasion [1,2,3,4] may, in fact, provide further support for the idea of using pUL56 as part of a vaccine strategy. Testing and comparing a live replicating *UL56*-deficient HSV-1 mutant to an adjuvanted recombinant pUL56 or *UL56* mRNA vaccine may significantly advance the HSV vaccine field by improving our knowledge of how immune responses against the virus are shifted through these different approaches.

Therapeutic HSV vaccines are considered alternatives to prophylactic approaches [32]. Viral reactivation leading to active disease in humans is associated with different degrees of immune suppression or deficiencies. While, for example, stress may lead to mild disease such as cold sores, more extensive immune suppression, as seen in HIV and transplant patients, can lead to frequent and severe disease manifestations. Furthermore, mutations in certain genes, e.g., TLR3 and TBK1, increase the risk of viral encephalitis [33]. For many of these patients, therapeutic vaccines could offer hope of preventing pain and potential severe complications. However, an added challenge for the development of an efficacious vaccine is the fact that these patients are immune-suppressed and thus will have a diminished capacity to mount vaccine-induced immune responses. Research is needed to better understand how immune imbalances and deficiencies impact patients’ HSV immune status and ability to respond to different types of HSV vaccines. Strategies that take advantage of insight into host immune responses to HSV and how HSV evade the mammalian immune system may have a greater chance of success. While our studies here revealed strong immune responses against a mutant HSV lacking *UL56*, further research is needed to understand which branches of the immune system are engaged by the used ΔUL56 mutant here and, more importantly, how pUL56 modulates humoral and cellular immunity in vivo. In this context, it should be noted that a therapeutic *UL56* mRNA vaccine could lead to further immune suppression by reducing cytokine responses and IFN-I production.

It is well established that many immune responses are stronger in women than men and that this sexual dimorphism is conserved in mammals [34]. While this is most commonly used to explain the higher incidence of many autoimmune diseases in women, it also has implications for vaccine responses [35]. Typically, women generate higher antibody titers than men, and titers decline more rapidly in men [35]. Here, we find that female mice are better protected against a lethal challenge with HSV-1 than male mice 6 months after receiving the ΔUL56 mutant virus (Figure 6). This effect could be due to an initial stronger response to the mutant virus in the female mice, but it may also be driven by a more rapid decline in the males. As both antibodies and T cells may be critical for HSV-1 control [36], future research could focus on examining levels of, for example, glycoproteins D (gD) and B (gB) antibodies and T cells, respectively, as indicators of immune engagement and durability. Since there is evidence that men experience more recurrences of active skin and mucosal HSV infections than women [37], it is important that sexual dimorphism of the immune response is carefully considered when developing HSV vaccines.

Analyses of gD-directed antibodies and gB-specific T cells may also shed light on how pUL56 suppresses the adaptive immune system. One immune mechanism that is known to be inhibited by UL56 is DNA sensing by cGAS [2]. This interaction is important for the induction of IFN-Is [38], which, in turn, may regulated both innate and adaptive immune pathways [18]. We observe clear differences in the infectivity of the ΔUL56 mutant and revertant strains in vivo (Figure 2) but not in vitro in keratinocytes (Figure 1). This could implicate adaptive immune responses as the most physiologically significant target for pUL56. However, further studies examining the role of UL56 in different cell types is needed to define UL56 function at the cellular level. A better understanding of how this protein affects both innate and adaptive immune pathways in vivo may especially benefit patients who are immune-suppressed.

## 5. Conclusions

While researchers in both academia and the industry have tried extensively to develop effective vaccines against HSV, none have made it into the clinic [29]. Compared to approaches involving killed viruses or recombinant proteins, live vaccines have the advantage of stimulating T cells in addition to neutralizing antibodies. The advent of mRNA vaccines has allowed more controlled approaches to simulate viral infections by expressing viral proteins within cells. As our insight into host immune responses against HSV and how the virus evades engagement of these improves, including the role of *UL56,* it should become possible to develop prophylactic or therapeutic vaccines against HSV.

## Figures and Tables

**Table 1:** Primers used for genotyping mice.

Gene	Primer	Sequence (5’–3’)	Target Direction
*Il1rap*	oIMR6916	CTT GGG TGG AGA GGC TAT TC	Mutant Forward
	oIMR6917	AGG TGA GAT GAC AGG AGA TC	Mutant Reverse
	9084	ACT ACA GCA CTG CCC ATT CC	Wildtype Forward
	oIMR4232	TGT AAT TGC CCG TGT CAT TG	Wildtype Reverse
*Il1rl2*	Il1rl2_comF	GGG CTA TTT TAT GGT CCA AAA CTA CCA G	Common Forward
	Il1rl2-wtR	GCT CTG GTC AAT GTG AAA TTG CAT TTA	Wildtype Reverse
	Il1rl2_mutR	ACC TCT CTC TGT AAG CTG GTC TGG G	Mutant Reverse

## Data Availability

The original contributions presented in this study are included in the article and Supplementary Material; further inquiries can be directed to the corresponding author.

## Supplementary Materials

The following supporting information can be downloaded at https://www.mdpi.com/article/10.3390/vaccines12080837/s1, Data file: Data Tongmuang Vaccines 2024.
